# Applying Digital Technology to Understand Human Experiences of Climate Change Impacts on Food Security and Mental Health: Scoping Review

**DOI:** 10.2196/54064

**Published:** 2024-07-23

**Authors:** Jasmin Bhawra, Nadine Elsahli, Jamin Patel

**Affiliations:** 1 CHANGE Research Lab, School of Occupational and Public Health Faculty of Community Services Toronto Metropolitan University Toronto, ON Canada; 2 School of Health Studies Faculty of Health Sciences Western University London, ON Canada

**Keywords:** climate change, digital health, ecoanxiety, environmental hazards, food security, mental health, scoping review, smartphone apps, digital apps, mobile health, mobile phone

## Abstract

**Background:**

The global impact of climate change ranges from intense heatwaves to extreme weather events that endanger entire ecosystems and people’s way of life. Adverse climate change events place undue stress on food and health systems, with consequences for human food security and mental health status. Ubiquitous digital devices, such as smartphones, have the potential to manage existing and emerging climate-related crises, given their ability to enable rapid response, instant communication, and knowledge sharing.

**Objective:**

This scoping review aimed to identify digital apps being used to capture or address climate change impacts on food security and mental health to inform the development of a digital citizen science initiative.

**Methods:**

A scoping review was conducted using 3 peer-reviewed databases (PubMed, IEEE Xplore, and Web of Science) and manual gray literature searches of relevant organizational (ie, governmental and nonprofit) websites to identify articles and reports published between January 2012 and July 2023. Three separate searches were conducted in each database to identify digital apps focused on climate change and (1) food security, (2) mental health, and (3) food security and mental health. Two reviewers conducted initial screening, with a third reviewer resolving any discrepancies. Articles focused on climate change impacts on wildlife or agriculture (ie, not human food security) were excluded. Full-text screening was conducted for shortlisted articles, and a final data abstraction table was generated, summarizing key app features, contextual factors, and participant involvement.

**Results:**

From the 656 records screened, 14 digital apps met the inclusion criteria. The food security apps (n=7, 50%) aimed to capture traditional knowledge to preserve food systems, conduct food security assessments, and aid users in decreasing food insecurity risk. The mental health apps (n=7, 50%) assessed climate change–related stress and provided users with coping strategies following adverse weather events. No digital apps examined the intersection of climate change, food security, and mental health. Key app features included user-to-user communication (n=5, 36%), knowledge databases (n=5, 36%), data collection and analysis (n=3, 21%), gamification (n=1, 7%), and educational resources (n=2, 14%) to address climate change impacts on food security or mental health. In total, 3 approaches to participant involvement were used across studies, including contributory (n=1, 7%), collaborative (n=1, 7%), and cocreative (n=1, 7%) approaches, to ensure the relevance and use of digital apps.

**Conclusions:**

Most digital apps identified provided a service to citizens to either prevent adverse climate change–related health impacts or manage these effects following an acute event or a natural disaster. The capacity of ubiquitous digital tools to enable near real-time communication, the involvement of various stakeholder groups, and their ability to share relevant educational resources in a timely manner are important for developing tailored climate change adaptation and mitigation strategies across jurisdictions.

## Introduction

### Background

Climate change has been identified as one of the biggest health crises of our time [1-3]. Numerous countries and governmental agencies have committed to various targets to combat climate change as part of the Paris Agreement amid profound global impacts ranging from intense heat and poor air quality, to extreme weather events that endanger entire ecosystems and people’s way of life [4-10]. Research has identified a myriad of direct and indirect risks that climate change has posed to human health, well-being, and, ultimately, survival in the 21st century [2,5,8,10-12].

Among the many impacted systems, evidence clearly demonstrates the effects of climate change on food systems, given the sensitivity of agriculture to weather conditions. Climate change has impacted not only the frequency and severity of extreme weather events, but also the timing and length of seasons, precipitation, and temperature [7,9,13,14]. These weather conditions have a direct relationship with growing seasons, soil fertility, nutrient bioavailability, pest resistance, and crop yield, which ultimately affect the food supply [13-15]. As a result, human food security is directly connected to climactic conditions and the global food supply, with people living in geographically vulnerable areas (ie, rural, remote, coastal, or northern arctic regions) [16-18] and those whose livelihoods are closely connected to the land (ie, farmers and Indigenous communities) experiencing the greatest risks to their food security [9,14,19,20]. The COVID-19 pandemic reminded the world of the global interconnectedness of its food supply [21], as it affected all populations—and not only those previously deemed to be living in areas geographically vulnerable to adverse climate change events [22-24]. Increasing frequency of extreme weather events [24] also directly affects mental health [21,22], with the term *solastalgia* coined to refer to the specific mental distress caused by environmental degradation and climate change [25]. Solastalgia has become increasingly prevalent in regions experiencing adverse weather events, especially where there is a lack of support or dedicated adaptation strategies [8].

The specific mechanisms linking climate change and mental health are still being explored [26]; however, the occurrence of specific climate change–related events (ie, extreme heat waves and flooding) has been found to increase stress, headaches, psychiatric hospitalizations, posttraumatic stress disorder, and suicide rates, particularly among patients with preexisting mental health conditions and among climate refugees who have been displaced [26-33]. In addition to the direct impacts of climate change on mental health, studies have also shown indirect impacts of climate-related events, such as heatwaves, on human behavior, including increased aggression and criminal activity [34-36], as well as low mood and impaired cognitive functioning resulting from heat stress [34,37,38]. Overall, adverse mental health impacts are worse among populations experiencing vulnerability, as they often lack control over climate change adaptation and mitigation strategies [39,40]. Other indirect effects of climate change on mental health could potentially result from experiences of climate change–related food insecurity [25,41,42]. Food insecurity research to date describes the experience as not just a physical health issue (ie, increasing the risk of malnutrition and nutrient deficiencies) but also a significant mental health challenge [17,41-44]. Families struggling with food security commonly report stigma, with significant shame, anxiety, stress, and even depression associated with not being able to access or afford adequate amounts of food [45-47]. Thus, in the current global scenario of exacerbated food insecurity connected to climate change, it is necessary to consider the combined experience of mental distress from both direct and indirect climate change impacts. Several studies exploring this important connection have emerged [48,49]; however, there is still a significant dearth of evidence in terms of the long-term effects of climate change on mental health through its detrimental impact on human food security.

Capturing the intersection of these complex global health issues requires leveraging existing and emerging technology [50-52]. Digital apps have long been used to track changing weather patterns and climate events [40,53], but the increasing frequency and severity of climate change warrant citizen engagement and participation to better understand both specific impacts and targeted solutions in the age of climate emergency [1,5,12]. Citizen science has a longstanding history of using citizen engagement to capture local environmental issues. It has proven to be particularly useful in tracking biodiversity loss [54,55], monitoring marine litter [56,57], and measuring air pollution levels across jurisdictions [58,59] for a deeper understanding of the ecological landscape.

Research has shown that digital technology can enhance early warning and emergency response systems while fostering citizen engagement for community health issues [35]. For instance, research-based digital apps, such as “Siaga Bencana” and the “Kanazawa and Kōchi Disaster Preparedness System,” leverage technology to assist individuals in areas prone to natural disasters [36,37]. Moreover, apps such as “InaRisk Personal” use innovative tools, such as cartographic visualization, to help improve knowledge of environmental hazards among youth [38]. Despite these advances, there is limited evidence of mobile apps focusing on food insecurity or mental health in a climate emergency.

Over the past decade, there has been an influx of digital apps focused on addressing mental health disorders in general [60-64] or household food security [26], which include a variety of interactive features, such as gamification [63] and digital chatbots [64], to manage anxiety and [26] connect users to food assistance programs [65]. Rapid advancements in the field of mobile apps show the prominence of ubiquitous digital devices for addressing a range of issues requiring effective monitoring and management, including climate change and health-related crises [50,52,66].

However, a key gap persists when we consider digital resource access issues among vulnerable communities, particularly those in the global south, which are also facing the brunt of adverse climate change impacts [23,40,66-68]. This gap, referred to as the *global digital divide*, is characterized by the “first-level” digital divide (ie, differences in digital access among citizens) and the “second-level” digital divide (ie, differences in citizens’ use of computers and the internet) [69]. The global digital divide can be attributed to various factors. First, smartphone penetration varies across the global south. Smartphone ownership in countries such as India, South Africa, Ghana, and Nigeria is very high [70], whereas there is relatively low access in Tanzania [71] and the Central African Republic [72]. Hence, the issue with access to digital apps is not simply a factor of smartphone affordability, as most of the global population owns smartphones. Instead, the issue of *internet inequity* [73,74] plays a larger role, as not all populations have affordable or easy access to the internet. The lack of internet access may thereby prevent citizens’ participation in digital research or global climate change initiatives. Moreover, differences in digital literacy levels among vulnerable sociodemographic groups can further the second-level digital divide and potentially deepen existing inequalities [75]. For instance, older individuals [76] and people with cognitive disabilities or communication challenges [77] often face barriers to using digital technologies effectively. Limited digital literacy skills among these groups can hinder their ability to fully participate in the digital economy, access essential services, and engage with digital tools for addressing pressing challenges such as climate change [75,76,78].

### Objectives

Despite advances in citizen science [79-81] and the application of digital technologies globally, no study to date has examined how or whether digital tools are addressing the intersection of climate change, food security, and mental health. Thus, this scoping review aimed to identify digital apps being used to capture and address human experiences of climate change impacts on both food security and mental health to inform the development of a digital citizen science initiative.

## Methods

### Search Strategy

This scoping review followed the PRISMA-ScR (Preferred Reporting Items for Systematic Reviews and Meta-Analyses Extension for Scoping Reviews) guidelines (Multimedia Appendix 1). The search strategy included a search of peer-reviewed and gray literature to answer the following research questions: (1) What digital apps are available to capture and understand human experiences of climate change impacts on food security? (2) What digital apps are available to capture and understand climate change–related impacts on mental health? and (3) What digital apps are available to capture and understand climate change impacts on human experiences of both food security and mental health? In total, 3 peer-reviewed databases were searched, including PubMed, a health and medical database; Web of Science, a sciences, social sciences, arts, and humanities database; and IEEE Xplore, an established computer science and engineering research database, to capture a broad range of studies involving digital technologies. The search strategy was developed in consultation with a university librarian and tailored for each database. Searches were organized into three categories, including (1) climate change + food security (CC+FS); (2) climate change + mental health (CC+MH); and (3) climate change + food security + mental health (CC+FS+MH), with all topic categories searched alongside terms for “digital apps” (Textbox 1). Various combinations of these search terms were used to conduct manual gray literature searches on Google and relevant organizational websites (ie, governmental and nonprofit organizations focused on climate change, public health, and digital health) to identify organizational reports and webpages not published in the peer-reviewed literature. All searches were conducted between November 7, 2022, and January 22, 2024. The search was limited to the past 10 years because while digital technology existed before 2012, the field of digital apps is rapidly evolving. For instance, many sensors that are commonplace in smartphones now were not available 10 years ago, including environment temperature and humidity sensors, which were released in 2013 and 2015, respectively [82].

**Comprehensive list of topics and search terms used in the scoping review literature search process.**

- Climate change
  Climate change, climate crisis, environment, global warming, heatwave, biodiversity loss, greenhouse effect, flood, drought, natural disaster, and emergency weather
- Food security
  Food security, food insecurity, food sovereignty, food supply, food shortage, food vulnerability, food scarcity, and food system
- Mental health
  Mental health, mental disorder, mental illness, depression, anxiety, ecoanxiety, solastalgia, and stress
- Digital apps
  App, web app, web application, mobile application, mobile app, phone application, phone app, digital platform, smartphone app, smartphone application, digital app, and digital application

### Inclusion Criteria

The inclusion and exclusion criteria are shown in Textbox 2. Peer-reviewed literature from all fields or disciplines covered by PubMed were included in the search. Web of Science searches were limited to the Science, Social Sciences, and Arts & Humanities Citation Indexes. IEEE Xplore searches were limited to journal articles. Titles and abstracts were shortlisted if they included search term or terms from each search topic category and mentioned the design, development, or use of digital platforms, apps, or tools. Only relevant articles published in 2012 or later (ie, over the past decade) were included because technology has rapidly evolved in this sector. This time filter was also used in the gray literature searches.

**Inclusion and exclusion criteria used in the scoping review literature search process.**

**Inclusion criteria**

- Article type
  Peer-reviewed journal articles
- Language
  English
- Publication year
  January 2012 to July 2023
- Study discipline
  Science
  Social sciences
  Arts and humanities
  Computer sciences
  Engineering
- Study focus
  Studies focusing on climate change and mental health; climate change and food security; or a combination of climate change, mental health, and food security
  Studies focusing on the design, development, or use of digital platforms, apps, or tools

**Exclusion criteria**

- Article type
  Literature reviews
  Protocol papers
- Language
  Any language other than English
- Publication year
  2011 or earlier
  July 2023 onward
- Study focus
  Studies that did not focus on the impacts of climate change on human food security experiences or mental health
  Studies that focused on climate change impacts on agriculture or farming practices
  Studies that focused on mental health impacts of the built environment (ie, neighborhood design and greenspaces) rather than impacts of climate change–related events

### Exclusion Criteria

Literature reviews and protocol papers, as well as articles not published in English or published earlier than 2012, were excluded. Articles that did not focus on the impacts of climate change on human food security experiences or mental health were excluded. For food security–specific searches, articles that focused on climate change impacts on agriculture in general (ie, not human food security) as well as farming practices or wildlife were excluded. For mental health–specific searches, digital apps that focused on mental health impacts of the built environment (ie, neighborhood design and greenspaces) rather than impacts of climate change–related events were excluded.

### Data Extraction and Appraisal

Overall, 2 reviewers (NE and JP) conducted a total of 3 searches corresponding with each research question. All citations were uploaded to the Zotero reference management software (Corporation for Digital Scholarship), and titles and abstracts were screened for relevance and fit with the inclusion criteria. A third reviewer (JB) helped to resolve any discrepancies, with web-based meetings held to reach consensus on article alignment with initial research questions and inclusion criteria. A consensus was reached on the final article shortlist (by JB, NE, and JP) after reviewing the full-text articles. A data summary table was created by extracting relevant data from the shortlisted articles and gray literature, including authors, year of publication, study objective, digital app features, areas of focus, and equity-related considerations.

## Results

### Overview

**Figure 1:**
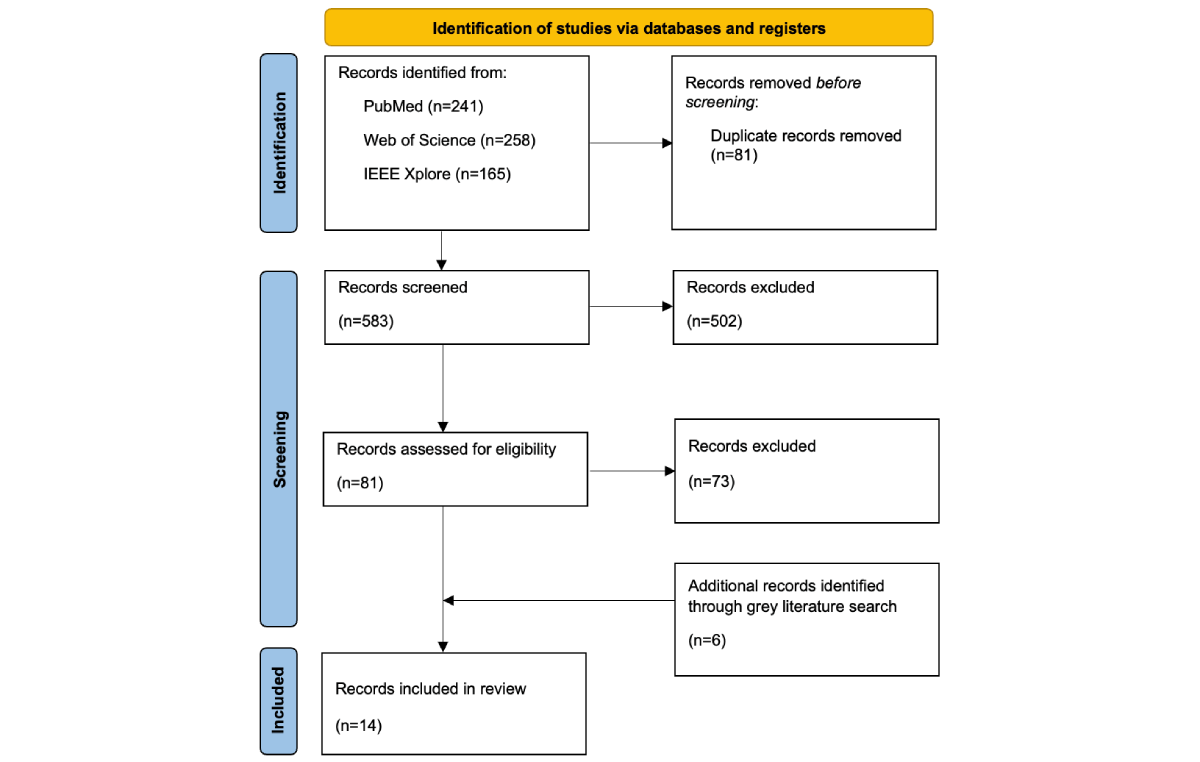
PRISMA-ScR (Preferred Reporting Items for Systematic Reviews and Meta-Analyses Extension for Scoping Reviews) flow diagram of the search and study selection processes.

As shown in Figure 1, a total of 656 citations were identified from 3 databases (CC+FS: n=393, 59.9%; CC+MH: n=233, 35.5%; CC+FS+MH: n=30, 4.6%). After removing duplicate records (n=81, 12.3%) and results that did not meet inclusion criteria at the title and abstract screening stage (n=394, 60.1%), 81 (12.3%) full-text records were reviewed. A total of 14 articles were selected, including 8 (57%) peer-reviewed articles (CC+FS: 3/8, 38%; CC+MH: 5/8, 62%; CC+FS+MH: 0/8, 0%) and 6 (43%) documents identified through gray literature searches.

Most articles identified focused on the development and evaluation of digital health apps for addressing various challenges and opportunities related to climate change. Studies with various study designs were reviewed, including observational cross-sectional studies (3/14, 21%), experimental studies (2/14, 14%), a development and validation study (1/14, 7%), a prospective cohort study (1/14, 7%), and an ethnographic study (1/14, 7%). Only 1 (7%) of the studies adopted a mixed methods approach, whereas the other studies used only qualitative methods (3/14, 21%) or quantitative methods (4/14, 29%). Studies were conducted in various countries across 3 continents, including Europe (3/14, 21%), Asia (1/14, 7%), and Africa (1/14, 7%). Of the 14 apps, 5 (36%) were web based, 6 (43%) were available on both iOS and Android, and 1 (7%) was available on Android only, and there was insufficient information provided by 2 (14%) studies to determine the operating system of the digital platform. Most digital apps (11/14, 79%) were publicly available and provided access links. The full data summary table is presented in Multimedia Appendix 2 [83-96].

### Climate Change and Food Security Apps

A total of 3 articles and 4 gray literature sources that captured climate change impacts on human food security were identified in this scoping review. In a study by Calvet-Mir et al [83], an app called the CONECT-e platform was used to enable the documentation, sharing, and exchange of traditional agroecological knowledge (TAeK) in Spain. By storing TAeK, the platform aimed to preserve culturally sensitive food systems to address food insecurity issues [83]. This process involved building a TAeK database and enabling communication within the app to facilitate collaborative discussions between knowledge users. Another study by Enenkel et al [84] used a smartphone-based digital app that conducted food security assessments in 101 households in the Central African Republic and shared findings with local aid organizations, such as Doctors Without Borders. The app used climate-focused features, including remote sensing of droughts, to determine the risk and prevalence of food insecurity in specific communities [84]. Another study conducted by Ramos et al [85] developed a web-based tool for food industry professionals to calculate the environmental impact of food production and to promote sustainable practices for the long-term stability of food supply chains. This involved creating a standardized data gathering system, selecting key environmental indicators, and establishing a methodology for a life cycle impact assessment.

From the gray literature sources, the following apps explored the impacts of climate change–related effects on human food security: Your Virtual Cold Chain Assistant, Good Empire, Floop, and Olio [86-89]. Good Empire, Floop, and Olio emphasized personal ownership and empowered users to make incremental behavioral adjustments to tackle climate change and food insecurity simultaneously [87-89]. These apps included features that allowed users to monitor and share their progress with others to highlight the positive changes they were making. In particular, Olio aimed to reduce food waste and curb overconsumption [89]. Good Empire and Floop primarily focused on the food carbon footprint, guiding users to make decisions based on their environmental implications [87,88]. Good Empire aimed to free communities from hunger [87], whereas Floop promoted reducing food waste by outlining climate-friendly recipes and meal plans [88]. Unlike the other 3 apps that focused on individual empowerment and behavior change, Virtual Cold Chain Assistant focused on providing a tool for farmers to address food sustainability and security while reducing carbon emissions in real time [86].

### Climate Change and Mental Health Apps

This scoping review identified 5 articles and 2 gray literature sources examining climate change impacts on mental health. Of the 5 articles, 3 (60%) focused on adults [90-92]; 1 (20%) focused on children and youth [93]; and 1 (20%) focused on a specific adult population of behavioral health responders, professionals who help individuals with mental and behavioral health conditions [94]. Most studies aimed to provide tangible mental health resources, including tips for managing mental health, links to best practice guidelines for health care providers, and information on nearby mental health treatment facilities or strategies to help those who may be struggling with their mental well-being due to climate change events [92-94]. Other apps assessed users’ behavior, feelings, and overall well-being after the delivery of climate-related warning messages (eg, “Warning: Thunderstorms with hurricane winds up to 120 km/h, as well as heavy rain with precipitation amounts around 35 l/m² will occur quickly”) [90] or self-reported data on user well-being in relation to temperature changes [91].

The gray literature sources identified 2 apps that addressed both climate change and mental health, Eco-Anxious and Climate Awakening [95,96]. These apps not only have a shared focus on creating a sense of community for sharing emotions elicited by climate change, but also focused on transforming these emotions into meaningful connections and learning how to have better climate-related conversations. For example, these apps included narrative storytelling, small-group sharing, and listening sessions, which enable users to connect, share experiences, and learn how to better manage climate-related emotions together.

### Climate Change, Food Security, and Mental Health Apps

There were no peer-reviewed articles or gray literature identified that addressed the third research question focused on digital apps that capture concurrent climate change impacts on food security and mental health.

### Features of Climate Change Apps

Common features of digital apps were identified (Table 1), with some apps having up to 3 features simultaneously. Across the 14 digital apps identified within this scoping review, the most common feature, seen in 6 (43%) apps, was the ability to track users’ perceptions, feelings, or health impacts related to climate change. Several digital apps (n=5, 36%) prioritized user-to-user communication within the app, whereas others (n=5, 36%) built a knowledge database related to the topic of climate change and food security or climate change and mental health. A few digital apps (n=3, 21%) analyzed the collected data to inform evidence-based findings, and 2 (14%) digital apps acted as educational apps consisting of guides or learning modules. Only 1 (7%) app (Good Empire) uniquely had the feature of gamification, with user contributions to the sustainable development goals (SDGs) being animated through a gamified process of unlocking statuses, achievements, and real-world rewards [87].

**Table 1:** Summary of technological features used across climate change digital apps identified in the scoping review (January 2015 to July 2023; N=14).

Study and year	Technological feature^a^					
	Tracking	User-to-user communication	Database	Analysis	Education	Gamification
Calvet-Mir et al [83], 2018		✓	✓			
Enenkel et al [84], 2015	✓					
Ramos et al [85], 2016				✓		
Seligman et al [94], 2015			✓		✓	
Tomczyk et al [90], 2021			✓			
Price et al [93], 2015					✓	
Bundo et al [91], 2023	✓					
Joshi et al [92], 2023	✓					
Your Virtual Cold Chain Assistant [86]	✓		✓	✓		
Good Empire [87]	✓	✓				✓
Floop App [88]	✓	✓		✓		
Olio [89]		✓				
Eco-Anxious Stories [95]			✓			
Climate Awakening [96]		✓				

^a^Features include tracking (monitoring and recording climate-related data), user-to-user communication (facilitating interactions and knowledge exchange), database (storage of climate-related information and resources), analysis (tools for data analysis and generating insights), education (offering climate change learning resources), and gamification (using interactive elements, ie, rewards and challenges, to engage users).

### Objectives and Uses of Climate Change Apps Across Contexts

All identified studies and gray literature sources addressed different aspects of climate change, food security, and mental health through their digital apps. Namely, of the 14 digital apps, 8 (57%) focused on providing services to citizens experiencing climate-related food insecurity [85-89] or mental health issues [94-96]. Other studies focused on collecting data related to the usability of the developed app (n=3, 21%) [83,84,93] or understanding the relationships between climate change and mental health (n=3, 21%) [90-92].

Various digital platforms were created and used within different contexts related to climate change. While some digital platforms (4/14, 29%) were developed to be used during or following natural disaster events [84,90,93,94], most identified digital platforms (10/14, 71%) were developed to be used as an ongoing preventive tool for climate-related food insecurity [83,85-89] or climate change–related mental health issues [91,92,95,96].

In total, 3 distinct approaches to citizen participation were observed in this review: contributory, collaborative, and cocreative approaches [83,84,92,97]. In the contributory approach used by Enenkel et al [84], local community health workers contributed to food security assessments after undergoing a training session. The local community health workers collected food security data, which were used to assess the feasibility of data collection on socioeconomic vulnerabilities related to malnutrition, resource accessibility, and coping capacities across communities in the Central African Republic. In contrast, Calvet-Mir et al [83] used a collaborative approach to citizen science, which involved societal participation in the documentation and sharing of traditional ecological information and practices. In this study, the CONECT-e platform was used to capture TAeK and enable knowledge exchange between researchers and local community members [83]. In a study by Joshi et al [92], a cocreative approach to citizen science was used, in which experiences shared by local community members were used to co-design a research framework. Researchers and citizen scientists both participated in the coanalysis of research data in this study [92]. Each citizen engagement approach, from data collection assistance (contributory) to participation in information sharing (collaborative) to active involvement in research design and analysis (cocreation), was used to complement specific study objectives and engage at different stages. Shortlisted articles were reviewed to identify whether digital apps considered equity as part of their app development or implementation process. Equity considerations could include the mention of specific population groups (ie, those disadvantaged in the context of climate change impacts), access issues (ie, smartphone and internet access issues), or digital literacy concerns. Only 3 (38%) of the 8 peer-reviewed studies mentioned equity-related considerations. For example, Joshi et al [92] examined power dynamics related to socioeconomic status and social identity in their sampling approach by taking proactive measures to represent marginalized castes in India. In addition, they used both male and female data collectors to mitigate sociocultural barriers to female participation in their study. Enenkel et al [84] examined climate change impacts on food security in Kabo, Central African Republic, one of the world’s most vulnerable regions in the global south in terms of poverty, violent conflicts, and weak disaster resilience. Finally, Bundo et al [91] examined how individuals with psychiatric disorders may be differentially impacted by climate change in Switzerland.

## Discussion

### Connecting Climate Change With Food Security and Mental Health Impacts

This scoping review is the first to explore the use of digital apps for understanding the relationships among climate change, food security, and mental health. While one scoping review by Martin et al [65] summarizing the use of digital platforms for food security was identified, our review uniquely captured global advancements in digital apps for climate change impacts on human experiences of both food security and mental health.

Climate change is significantly impacting food systems globally, ranging from adverse effects of extreme weather events [34,98,99] to positive effects such as longer growing seasons [100,101] in some regions. Climate change has contributed to lower crop yields and food shortages in regions across Africa and Southeast Asia [18,41,102] while also impacting human food security in vulnerable arctic and coastal regions in the global north [34,99]. While no apps concurrently capturing climate change–related food security and mental health issues were identified in this review, research has shown that experiences of food insecurity are linked to stress, anxiety, and poor mental health outcomes in general [90,91,103-106] and can be exacerbated during climate emergencies [107,108]. In many instances, communities that are vulnerable to adverse climate change events are also disproportionately affected by food insecurity [34,109,110]. Consistent with existing literature [40,50-53], this review found that digital apps, particularly those that provide real-time alerts, information, and communication, can not only ensure that solutions are catered to communities’ specific needs, but also foster a sense of empowerment among community members [83,84,92].

### The Use of Digital Apps for Climate Change–Related Food Insecurity

This scoping review uncovered a few digital apps focused on climate change impacts on human food security [83-89]. These apps assessed food security status and food acquisition patterns to support users in engaging in sustainable food practices. There was a large focus on individual or community behavior change to enhance food security, with one app focused on exchanging traditional cultural knowledge to preserve food systems [83]. Food security involves not only access to and affordability of adequate food, but importantly also includes culturally appropriate food and acquisition practices—particularly in Indigenous and ethnic communities. [67,111]. Thus, the availability of digital tools to capture and share traditional ecological knowledge is an important aspect of inclusive and user-centered climate change apps, as cultural aspects are often not captured in many food security assessments [68]. Given their focus on behavior change, most digital apps identified were designed for use within communities (ie, user-to-user interaction) and, therefore, had limited connection to other stakeholder groups (eg, decision makers and food assistance programs).

Many food security–focused digital apps identified in this scoping review relied on user input and activity to understand specific climate change impacts. These platforms often used crowdsourced data, where users contributed information and shared experiences. In one study by Enenkel et al [84], which implemented a digital app in communities within the Central African Republic, one of the most vulnerable regions in the global south, user contributions enabled a better understanding of food security in 101 households. This user-driven approach increased the diversity and potential accuracy of data, enhancing the digital platform’s ability to address local challenges effectively. Moreover, the digital apps identified in this scoping review empowered users by implementing citizen-based data collection and data-driven output to users, including offering advice and suggestions for sustainable alternatives to food consumption and production. For example, Calvet-Mir et al [83] and Ramos et al [85] examined climate change and food security apps focused on empowering farmers and food producers to make informed decisions to address adverse climate change impacts. By collecting and analyzing data on farming and food production practices, these digital platforms provided content personalized to their industries and geographic locations. Similarly, other climate change and food security apps that focused on behavior change included features such as progress monitoring to empower users to engage in more sustainable practices that address food security [87-89]. These approaches prioritized local knowledge, empowerment, and users’ control in shaping their own food systems [111,112].

While digital apps can provide near real-time information about food insecurity risk, our scoping review found limited evidence of the evaluation of these apps. Similarly, a previous scoping review examining the use of digital platforms to provide food assistance found a gap in the evidence on the effectiveness and impact of these tools [113]. In this review, we found only 1 study, by Enenkel et al [84], that conducted an evaluation to assess app usability following a food security intervention where the authors calculated the number of smartphone-based food security assessments that could be performed within a 6-hour working day. The lack of evaluation activities can be attributed, in part, to the fact that many apps are created in the commercial rather than research domain [113]. Moreover, existing platforms are still in the early stages of implementation due to the relative recency of their development between 2015 and 2023 [113].

Despite the limited number of apps focused on human food security, there are promising advances in the use of digital technology and tools to assess and manage climate change impacts on agriculture and food security from a land management perspective [114-116]. Overall, there is potential to not only capture food security status using digital apps but also actively deploy targeted digital health interventions connecting communities with relevant organizations or decision makers to address existing food security issues [115].

### Connecting Climate Change to Mental Health Outcomes

In addition to examining the role of digital apps in climate change–related impacts on food security, this review also examined their role in mental health. These apps focused on providing users with mental health resources and strategies to cope with solastalgia. Some apps assessed users’ feelings following the delivery of climate change warnings or messages, while others focused on connecting users to each other to engage in climate-related conversations as a part of managing emotions or stress.

In this review, studies found that while adults experienced anxiety or stress related to climate change events [90-94], those experiencing greater vulnerability due to various social factors, including age, gender, and socioeconomic status, were disproportionately excluded from climate change initiatives [92]. The majority of recent research in this area has shown that in addition to being active participants in climate change activism [117,118], children and youth are particularly vulnerable to both climate change impacts [119-122] and mental health challenges [121]. A study conducted by Hickman et al [121] across 10 countries found that 45% of children and youths report that climate change–related anxiety negatively affected their daily life and functioning.

Eco-Anxious and Climate Awakening were digital apps identified in this review that included features for users of all ages to connect with each other to share their mental health status. These apps aimed to improve mental health and foster meaningful connections among app users by improving climate awareness; promoting conversations about climate-related emotions; and including web-based storytelling using guided frameworks and exercises [95], as well as climate-related prompts through small-group listening sessions [96]. Evidence has shown that sharing concerns about climate change can potentially reduce feelings of stress and anxiety, even in the absence of a specific intervention [123]. Most digital apps identified did not collect mental health data from users but instead addressed mental health concerns by providing resources and opportunities for users to connect. The studies that collected mental health data were designed to explore the intersection of climate change and mental health rather than improving mental health outcomes. For instance, Climate Awakening and Eco-Anxious [95,96] both used innovative approaches, including features for web-based storytelling and small-group sessions, to provide users with a sense of community by allowing them to share how climate change is impacting their mental health.

Similar to the apps uncovered for climate change and food insecurity, no studies identified in this review evaluated the impact of these digital platforms on mental health. This may be due to the relative recency of digital platforms examining both climate change and mental health [84]. Future studies should not only examine the influence of digital apps on addressing climate change impacts on mental health, but also compare their effectiveness across age groups and other sociodemographic groups to understand potential differences in uptake and use.

### Gaps in Addressing the Intersection of Climate Change, Food Security, and Mental Health

This scoping review did not identify any digital apps that simultaneously connected the concepts of climate change to both food security and mental health; however, key features identified in the shortlisted digital apps could be considered for this purpose. Given the interconnected and cumulative impacts of climate change on food systems and mental well-being [103-106], there is immense potential to address numerous SDGs (SDG 1: Zero Hunger; SDG 2: Good Health and Well-being; and SDG 13: Climate Action) through this holistic lens [124]. Specific app features described in this scoping review, including adverse weather event tracking, alerts, educational resources and support, and user-to-user communication, can importantly be used to promote rapid responses to the direct effects of climate change on (1) food insecurity and (2) mental health issues and (3) the indirect effects of climate change–related food insecurity on mental health across sectors. In particular, addressing the SDGs requires a concerted effort whereby citizens (ie, app users) track local impacts; decision makers provide citizens with timely information and resources; and, globally, there is an effort to share knowledge and resources toward these goals.

Our search uncovered a study protocol for the Food Equity and Environmental Data Sovereignty project, which was the only example of a digital app that aimed to address climate change, food security, and mental health concurrently [125]. The Food Equity and Environmental Data Sovereignty project involves a smartphone-based digital app to monitor, mitigate, and manage adverse impacts of climate change on food security and mental health among vulnerable communities. The project takes a digital citizen science approach to cocreate and share knowledge among user groups to enable near real-time monitoring; communication; and response to the interconnected issues of climate change, food security, and mental health [125]. Specific app features such as time- and location stamping specific environmental hazards or adverse weather events, citizen reporting of perceived impacts of climate change on local food systems and indicators of mental health (ie, stress and anxiety), and a digital dashboard aim to connect citizens to community decision makers.

Another key gap identified through this review was the lack of discussion of either access or equity-related issues in digital app design. It is necessary to factor the global digital divide, which includes unequal access to digital tools (ie, smartphones) and the internet, into digital app development and deployment for disadvantaged populations [69,73,74]. Out of the 14 studies identified, only 2 (14%) were focused on the global south, and these were the only ones that considered equity-related issues as either a specific barrier to or determinant of app use. For example, Joshi et al [92] created an app called SenseMaker to capture gendered experiences of climate change in Bihar, India. This study acknowledged existing differences in literacy levels, with the national literacy rate among women being 51.5%, compared to 71.2% among men, which influenced digital literacy, numerous health indicators, and the ability to participate in climate change–related research initiatives [92]. Thus, Joshi et al [92] focused on the co-design of a research framework and aimed to capture intersectional inequalities related to climate change vulnerability, a critical aspect of creating equitable climate action. Enenkel et al [84] conducted a study in the Central African Republic that importantly recognized that, “While droughts and their precursors (e.g. ocean temperature anomalies) can be monitored by satellites, the resulting socio-economic impacts...that are related to people’s vulnerabilities or coping capacities remain mostly hidden to the eye of a satellite.” The Central African Republic has relatively low access to smartphones [72], and although the authors did not discuss the global digital divide or specific issues of digital literacy, they noted that community health workers had to be trained before data collection, given their limited experience in using smartphones [84]. Ultimately, low digital literacy created specific challenges that could have impacted the uptake and use of the digital app (ie, entering data into the smartphone-based app and understanding how to navigate the app, including swiping vs tapping to access specific app functions) [84].

While the “digital divide” is a prominent issue in the global context, it is necessary to also consider the digital divide *within* countries, as certain population subgroups (ie, non–native language speakers and older adults) interact with and use technology in different ways, which could exacerbate disadvantages, particularly when essential health services or resources are being shared via digital means. It is important for digital apps to capture issues of digital literacy and internet access not only as potentially confounding factors (ie, which could affect app uptake and use) but also to improve reach to populations in an equitable manner.

### The Need for Digital Tools to Monitor, Mitigate, and Manage Climate Change Impacts

The digital apps uncovered in this scoping review importantly demonstrated a range of approaches to participant involvement in either app development or deployment, including contributory, collaborative, and cocreation approaches. Participant or user involvement was essential not only to ensuring the relevance of the digital apps to users’ needs but also, ultimately, to the uptake and use of the apps.

The use of mobile digital technology has the potential to enable continuous citizen involvement and equitable reach to geographically rural and remote communities, including vulnerable populations who may not have easy physical access to information or resources to manage adverse climate change impacts [126]. Citizen science has a history of engaging people to capture environmental data [54-59]. Thus, using digital citizen science (ie, citizen participation in studies or projects via digital means), there is potential to generate enormous amounts of big data from citizen-owned devices (ie, smartphones), which can transform not only how climate change events are surveilled but also the prediction and prevention of community health impacts related to food security and mental health [34,54-59,127]. Of the 14 studies identified in this scoping review, 3 (21%) used citizen science approaches. Calvet-Mir et al [83] applied a collaborative citizen science method, enabling user input to promote the collaborative documentation and sharing of traditional ecological knowledge and practices. Enenkel et al [84] trained community health workers familiar with the basics of food security assessments to conduct data collection within the community, showcasing a contributory citizen science approach that has the potential to significantly enhance the efficiency of food security assessments. Joshi et al [92] used a cocreative approach, allowing citizen scientists to lead the analysis of their own data, while researchers adopted a facilitative role, mitigating subjective biases. Despite the advantages of citizen science approaches, most of the studies reviewed did not use them.

In addition, the inherent complexity and interdisciplinarity of climate change impacts require the use of big data and a systems thinking lens that works across sectors (ie, food, health, environment, and justice) to address risks and opportunities [50,128,129]. Digital apps have the ability to reach a larger and more geographically diverse user base [130], as mentioned in the study by Calvet-Mir et al [83], where user engagement with the app included 150,000 app visits across 467 users 1 year after its launch. In this digital age, the use of innovative approaches such as ecological momentary assessments can play an important role, as they allow researchers to potentially capture the mental health status of populations with reduced recall biases compared to traditional retrospective surveys [131,132]. One study collected data from users to examine associations between climate change predictors and mental health outcomes [91] and used ecologic momentary assessments to allow users to self-report their mental health. Advanced processing techniques, such as remote sensing, can also be used to make predictions and identify trends in real time [133].

Overall, the digital apps identified in this scoping review were designed to provide citizens with a service, whether it was for use during or immediately following a climate change–related natural disaster or to be used for the preventive management of ongoing risks. Thus, there is immense potential for the use of digital technologies to mitigate, manage, and potentially prevent food insecurity and mental distress, particularly if these issues are addressed concurrently and holistically, given their interconnection.

### Limitations

This scoping review presents a comprehensive search of both peer-reviewed and gray literature, and the use of multiple databases enabled the capture of a range of literature across disciplines. Most apps shortlisted in this review were identified from literature published between 2015 and 2023, with 5 (62%) out of 8 apps uncovered from peer-reviewed literature published before 2019. The concentration of apps before 2019 may reflect the redirected focus of digital health research to the COVID-19 pandemic, thereby limiting work on climate change impacts on health [134-137]. This study excluded non–English language literature and, therefore, may have missed digital apps published in other languages. Moreover, this scoping review explored 3 distinct but interrelated research questions in an effort to identify digital apps for climate change impacts on both food security and mental health; however, we were unable to assess the quality of the shortlisted digital apps, given the recency of most apps and lack of publicly reported evaluation activities. There was no evidence of evaluations being conducted, which limits our ability to understand the potential impact these apps had on improving food security and mental health.

### Conclusions

This scoping review aimed to understand how digital apps have been used to capture human experiences of climate change impacts on food security and mental health. While some digital apps focused on food security or mental health, there were no apps identified that took a holistic approach to concurrently assess food systems and mental health outcomes associated with experiences of climate change. Participatory approaches were used to engage and involve app users, and a variety of app features were used to collect data, enable user communication about climate change–related events, and manage the risk of food insecurity or mental distress.

Given the frequency of adverse climate change–related events globally, there is an urgent need for future studies to comprehensively capture and evaluate the interconnections among food security, mental health, and climate change while considering communities’ local knowledge, concerns, and specific risk factors. By harnessing the power of digital apps, researchers and decision makers can partner with communities to effectively respond to and navigate climate change impacts on both food security and mental health. In particular, digital apps that enable near real-time alerts, communication, and potential for intervention can ensure that solutions are tailored to communities’ specific needs and challenges while fostering a sense of empowerment and ownership among citizens.

## Appendix

Multimedia Appendix 1 PRISMA-ScR checklist.

Multimedia Appendix 2 Scoping review data extraction table.

## Abbreviations

CC+FS: climate change + food security
CC+FS+MH: climate change + food security + mental health
CC+MH: climate change + mental health
PRISMA-ScR: Preferred Reporting Items for Systematic Reviews and Meta-Analyses Extension for Scoping Reviews
SDG: sustainable development goal
TAeK: traditional agroecological knowledge

## Data Availability

All data generated or analyzed during this study are included in this published article (and its supplementary information files).
