# Supplementary material for: Applying Digital Technology to Understand Human Experiences of Climate Change Impacts on Food Security and Mental Health: Scoping Review
Source: JMIR Public Health Surveill. 2024 Jul 23;10:e54064. doi: 10.2196/54064 (PMC11303902; doi:10.2196/54064)
Supplement: Multimedia Appendix 2 [file publichealth_v10i1e54064_app2.docx]

## Multimedia Appendix 1 - Scoping review data extraction table

**Summary of peer-reviewed studies using digital apps to examine the connection between climate change and food security identified in the scoping review.**

| **Authors, Year** | **Study Setting** | **Target Population** | **Study Objective** | **Methods** | **Digital App Name** | **Digital App Features** | **Operating System and Access** | **Areas of Focus** | **Equity Considerations** | **Key Findings** |
| --- | --- | --- | --- | --- | --- | --- | --- | --- | --- | --- |
| Calvet-Mir et al. (2018) | Spain | Traditional agroecological knowledge holders and users | Assess the potential of the CONECT-e platform as a tool to support the sharing and exchange of traditional agroecological knowledge | Qualitative  Observational cross-sectional study | CONECT-e Platform | Citizen science  Knowledge database  Discussion forum for knowledge sharing | Web-based app  Information regarding access not publicly available | Traditional agroecological knowledge (TAeK)  Indigenous health  Citizen science | Equity considerations not mentioned | The platform was able to facilitate sharing and exchange of traditional agroecological knowledge among users to help them adapt to the challenges posed by climate change |
| Enenkel et al. (2015) | Kabo, Central African Republic | Local community health workers and households | Evaluate the potential of mobile data collection and remote sensing to improve food security monitoring and early warning in the Central African Republic | Mixed-methods design (qualitative and quantitative)  Observational cross-sectional study | SATIDA COLLECT | Food security assessments  Remote sensing of drought indicators | Android  Free access (open source) | Food security assessment  Climate-related satellite-driven data and indicators  Malnutrition  Assessing socioeconomic vulnerabilities to climate change | Study focused on a country vulnerable to climate change impacts in the global south | Their platform enabled the collection of data related to food-security and use of remote sensing data to identify food insecure and vulnerable regions  The platform provided an efficient system for food security assessments |
| Ramos et al. (2016) | N/A | Professionals in the food industry | Develop and introduce a web-based tool for calculating the environmental impact of food products | Quantitative  Development and validation study | SENSE tool (web-based application) | User input related to food products  Environmental impact analysis | Web-based app  Information regarding access not publicly available | Food sustainability  Climate change  Water use  Land use | Equity considerations not mentioned | The platform provided an easy-to-use and accessible tool to help food industry professionals in small and medium enterprises with assessing their sustainability (i.e., calculating the environment impact of their products) |

**Summary of peer-reviewed studies using digital apps to examine the connection between climate change and mental health identified in the scoping review.**

| **Authors, Year** | **Study Setting** | **Target Population** | **Study Objective** | **Methods** | **Digital App Name** | **Digital App Features** | **Operating System and Access** | **Areas of Focus** | **Equity Considerations** | **Key Findings** |
| --- | --- | --- | --- | --- | --- | --- | --- | --- | --- | --- |
| Seligman et al. (2015) | N/A | Behavioural health responders | Promote mental health, prevent substance misuse, and provide treatments and supports to foster recovery from natural disasters | Qualitative  Observational cross-sectional study | SAMHSA Disaster Application | Resource kit including tip sheets and guides  Database of behavioural health service providers | iOS and Android  Free access (open source) | Natural disasters  Mental wellbeing  Health preparedness & disaster planning | Equity considerations not mentioned | Qualitative interviews with users revealed that sharing of resources survivors of natural disasters were important features to provide in real-time  The interviews also revealed that a review of key counselling skills and information was a valuable feature  Preloading mental health treatment locations was viewed as a valuable feature |
| Tomczyk et al. (2021) | Berlin, Germany | Adults affected by disaster | Explore affective reactions and behavioural intentions following app-based warnings for severe weather events | Quantitative  Experimental study | Local App | Database of location, sights, traffic, and weather | Information regarding platform and access not available | Natural disasters  Severe weather events  Anxiety level analysis | Equity considerations not mentioned | Their platform found that participants who received a warning message and were confronted with a thunderstorm showed the highest increase in momentary anxiety, which predicted information seeking intentions  The platform enabled app-based warning messages regarding severe weather events as well as the event itself, which differentially affected momentary anxiety (i.e., negative affect) |
| Price et al. (2015) | N/A | Adolescents affected by natural disasters | Evaluate usability of mental health intervention following natural disasters | Quantitative  Randomized controlled trial | Bounce back now | Knowledge modules | iOS and Android  Free access | Natural disasters  Mental wellbeing  Treatment access | Equity considerations not mentioned | The platform was able to reach and engage many adolescents affected by natural disasters to address mental health symptoms |
| Bundo et al. (2023) | Luasanne, Switzerland | Adults | Explore how increases in ambient temperature affects mood | Quantitative  Prospective cohort study | N/A | Self-reported mood EMA | Cellular phones (operating system not specified)  Information regarding access not available | Impact of rising temperature on mental health  Daily mood  Psychiatric disorders  Neuroticism | Study examined a population experiencing psychiatric disorders to understand differential impacts of climate change on mental health | The tool found that there is a higher likelihood of being in a positive mood with increasing temperatures  The tool revealed that individuals with certain psychiatric disorders have a higher likelihood of negative mood with increasing temperatures, indicating their altered response to heat |
| Joshi et al. (2023) | Bihar, India | Adult women | Assess the use of a digital ethnography tool to understand experiences, perceptions, and biases related to climate change related impacts | Qualitative  Ethnography | SenseMaker | Narrative data collection (experiences and perspectives) | Web-based application  Information regarding access not available | Resilience and coping strategies  Intersectional inequalities | Study incorporated power dynamics between socioeconomic status and social identity (i.e., caste and gender) in sampling strategy  Study took special measures to capture diverse voices of marginalized castes  Study involved both male and female data collectors to address socio-cultural barriers that inhibited females from providing their stories | The platform highlights how social factors influence marginality and vulnerability to climate change impacts  The platform presented that the most vulnerable groups, including marginalized women, may not be adequately included in climate change initiatives |

**Summary of grey literature on the use of digital apps for examining the connections between climate change, food security, and mental health identified in the scoping review.**

| **App Name** | **App Objective** | **App Features** | **Operating System and Access** | **Areas of Focus** |
| --- | --- | --- | --- | --- |
|  | | | | |
| Your Virtual Cold Chain Assistant | Minimize food waste among smallholder farmers through digital innovation | Uses machine learning and physics-based food modelling to provide smallholder farmers with actionable post-harvest and market intelligence  Users receive real-time information about when the produce in storage is reaching the end of life  App sensors capture data points such as temperature and humidity | Web-based application  Free access (open source) | Food sustainability  Cooling operations |
| Good Empire | Empower users to engage in more sustainable practices by gamifying their contributions to sustainability initiatives in their community | Visualize, monitor, and share your impact on challenges aligned with the Sustainable Development Goals (SDGs)  Unlock status, achievements, and real-world rewards | iOS and Android  Free access (open source) | Carbon emission  Plastic waste  Hunger and poverty  Empower women and girls |
| Floop | Reduce climate impact by promoting sustainable dietary habits that minimize the carbon footprint | Monitors meals consumed and associated carbon footprint  Uses life cycle assessment data to assess carbon dioxide emissions produced by meals  Outlines climate-friendly recipes and provides meal plans to reduce food waste | iOS and Android  Free access (open source) | Food sustainability  Food-carbon footprint |
| Olio | Empower communities to engage in practices that mitigate the climate crisis | Snap photos of unwanted items, message to arrange pick-up, and share your impact | iOS and Android  Free access (open source) | Food sustainability  Waste and overconsumption  Climate crisis |
|  | | | | |
| EcoAnxious | An online storytelling community to share ecoanxiety and transform anxiety into meaningful connections | Offers resources for mental wellness, anxiety tools and tips, and an eco-anxiety reflection guide | Web-based application  Free access (open source) | Climate change  Mental health  Storytelling  Equity |
| Climate Awakening | Connecting people through small group conversations to share and learn how to have better climate conversations | Creates small group sharing and listening sessions to discuss climate fears, rage, and grief | Web-based application  Free access (open source) | Climate change  Mental health |
